# The Predictive Value of Platelet‐to‐Lymphocyte Ratio Before and After Percutaneous Coronary Intervention in Patients With Acute ST‐Segment Elevation Myocardial Infarction: A Retrospective Cohort Study in Palestine

**DOI:** 10.1002/clc.70422

**Published:** 2026-07-27

**Authors:** Anwar Zahran, Saja Amer, Mohammad Bdair, Seema Hameedi, Roa Badran, Mays Safa, Abdelfattah M. Dahmas, Ahmad Mohammad, Abdul‐rahman Abdel‐karim, Zaher Nazzal

**Affiliations:** ^1^ Department of Medicine, Faculty of Medicine and Allied Medical Sciences An‐Najah National University Nablus Palestine; ^2^ Hurley Medical Center‐MSU Flint Michigan USA; ^3^ An‐Najah National University Hospital Nablus Palestine

**Keywords:** heart failure, platelet‐to‐lymphocyte ratio, PLR, primary PCI, STEMI

## Abstract

**Background:**

Inflammation and thrombosis influence short‐term outcomes after primary percutaneous coronary intervention (PCI) for acute ST‐segment elevation myocardial infarction (STEMI). The platelet‐to‐lymphocyte ratio (PLR) is an inexpensive marker that may support bedside risk stratification.

**Objective:**

To assess whether pre‐PCI and post‐PCI PLR predict in‐hospital outcomes and 90‐day all‐cause mortality in STEMI patients undergoing primary PCI in Palestine.

**Methods:**

This retrospective cohort study included STEMI patients referred for primary PCI at An‐Najah National University Hospital. Of 413 eligible patients, 118 were excluded for incomplete records, leaving 295 patients for analysis. PLR was measured before and after PCI and dichotomized using ROC‐derived cutoffs: pre‐PCI PLR < 21.68 versus ≥ 21.68 and post‐PCI PLR < 14.85 versus ≥ 14.85. Outcomes were compared between groups, and multivariable logistic regression was used to assess independent associations.

**Results:**

Elevated pre‐PCI PLR was associated with greater in‐hospital clinical instability and remained independently associated with the composite in‐hospital clinical endpoint, both per 10‐unit increase (OR 1.41, 95% CI 1.13–1.74; *p* = 0.002) and using the cutoff ≥ 21.68 (OR 2.90, 95% CI 1.55–5.44; *p* < 0.001). However, pre‐PCI PLR was not independently associated with heart failure or 90‐day mortality. Post‐PCI PLR showed stronger prognostic value and was independently associated with composite in‐hospital events, heart failure, and 90‐day mortality, both as a continuous variable and using the cutoff ≥ 14.85.

**Conclusion:**

Post‐PCI PLR demonstrated stronger prognostic value than pre‐PCI PLR and may serve as a simple, low‐cost adjunctive marker for short‐term risk stratification after primary PCI in STEMI patients.

## Graphical abstract

Post‐PCI platelet‐to‐lymphocyte ratio showed stronger prognostic value than pre‐PCI PLR in STEMI patients undergoing primary PCI. Elevated post‐PCI PLR independently associated with composite in‐hospital clinical events, heart failure, and 90‐day mortality, supporting its role as a simple adjunctive marker for short‐term risk stratification.

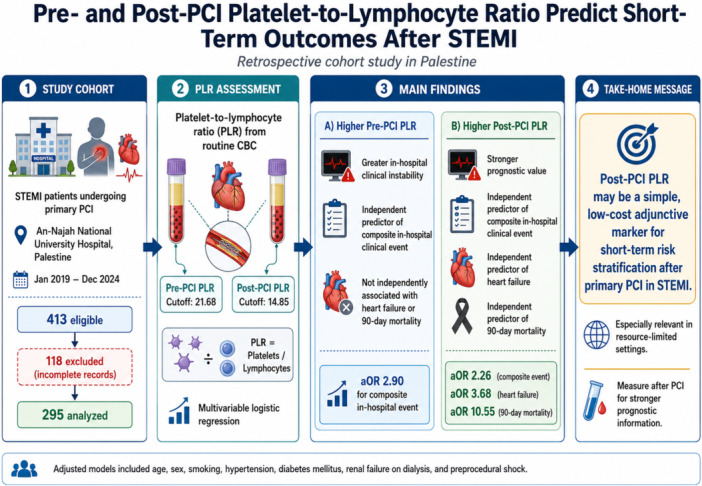

**Abbreviations**
ACS: acute coronary syndrome
BMI: body mass index
CABG: coronary artery bypass grafting
CBC: complete blood count
CHF: congestive heart failure
CVA: cerebrovascular accident
D2B: door‐to‐balloon
IQR: interquartile range
LVEF: left ventricular ejection fraction
MACE: major adverse cardiovascular events
NYHA: New York Heart Association
PCI: percutaneous coronary intervention
PLR: platelet‐to‐lymphocyte ratio
ROC: receiver operating characteristic
SMD: standardized mean difference
STEMI: ST‐segment elevation myocardial infarction

## 1 Introduction

Acute ST‐segment elevation myocardial infarction (STEMI) is a life‐threatening manifestation of acute coronary syndrome (ACS) [1], typically caused by rupture of an atherosclerotic plaque and subsequent thrombus formation. The standard therapy for STEMI is urgent revascularization, usually by primary percutaneous coronary intervention (PCI), which aims for rapid restoration of coronary blood flow to minimize myocardial damage [2]. Despite advancements in reperfusion techniques, STEMI patients who underwent PCI remain at risk of adverse events such as heart failure, arrhythmias, end‐organ damage, and death [3].

The pathophysiology of STEMI involves a complex interaction between inflammation, thrombosis, and immune dysregulation [4]. Platelets play a central role in thrombus formation, while lymphocytes are key regulators of the immune response [5, 6]. Inflammatory markers have increasingly been studied as tools for early risk stratification [7, 8, 9, 10, 11]. One such marker is the platelet‐to‐lymphocyte ratio (PLR), derived from a routine complete blood count (CBC) test, which reflects both thrombotic activity (platelet count) and inflammatory status (lymphocyte count). PLR has been proposed as a promising, inexpensive, and easily accessible prognostic indicator in various cardiovascular diseases, including STEMI [12, 13].

Recent studies suggest that a high PLR is associated with worse clinical outcomes, such as the no‐reflow phenomenon, larger infarct size, and increased rates of major adverse cardiovascular events (MACEs) [14, 15]. However, the findings remain controversial across different populations and settings [16]. In resource‐constrained settings such as Palestine, the clinical burden is even greater due to healthcare disparities, delayed hospital presentations, and limited post‐PCI monitoring capabilities.

This study aimed to evaluate the predictive value of the PLR measured before and after PCI, in patients with acute STEMI in Palestine and its association with in‐hospital clinical outcomes and 90‐day mortality, to support better risk assessment and outcomes.

## 2 Methodology

### 2.1 Study Design

We conducted a retrospective cohort study to evaluate the associations of pre‐PCI and post‐PCI PLR with clinical outcomes among patients with STEMI undergoing primary PCI.

### 2.2 Population

Our study population was all STEMI patients who were referred to An‐Najah National University Hospital for primary percutaneous coronary intervention between January 2019 and December 2024. Patients with incomplete data records were excluded.

### 2.3 Sample Size and Sampling Method

This retrospective study included all eligible patients who underwent primary PCI during the predefined study period. A total of 413 patients were screened, of whom 118 were excluded because of incomplete records, leaving 295 patients in the final cohort. Complete data for calculating pre‐PCI PLR were available for 272 patients, while complete post‐PCI PLR data were available for 249 patients. Analyses were conducted using available‐case data for each PLR measurement and outcome.

### 2.4 Study Variables

We examined outcomes in relation to a prespecified set of patient‐level covariates. These included demographics (age, sex, BMI), cardiovascular risk factors (smoking, hypertension, diabetes, dyslipidemia), prior cardiovascular history (previous MI/PCI/CABG, heart failure, cerebrovascular disease, renal failure), baseline clinical condition (including preprocedural shock, preprocedural LVEF, and baseline laboratory measures). Procedural features and intra‐ or postprocedural complications were documented according to the definitions in the study protocol. A full summary of all collected variables and their operational definitions is presented in Supporting Information S1: Table 1.

PLR was calculated by dividing the platelet count by the absolute lymphocyte count, with both values expressed in the same units, from routine complete blood count testing. Pre‐PCI PLR was calculated from the most recent complete blood count obtained before PCI. Post‐PCI PLR was calculated from the first available complete blood count obtained after PCI during the index hospitalization.

The primary aim was to assess the relationship between PLR (the main independent variable) and the risk of any in‐hospital adverse event during the index admission. Secondary aims were to evaluate specific in‐hospital complications, hospital length of stay, and 90‐day mortality. The composite in‐hospital clinical outcome was defined as the occurrence of at least one of the following during the index hospitalization: acute congestive heart failure, life‐threatening arrhythmia, mechanical ventilation, circulatory support, cardiogenic shock, heart failure, cardiac tamponade, cerebrovascular accident/stroke, postprocedural dialysis, intra‐procedural mortality, CABG during admission, bleeding within 72 h, vascular complications requiring treatment, or red‐blood‐cell/whole‐blood transfusion. Mortality was defined as death from any cause within 90 days of the index PCI. Because the exact date of death or time from PCI to death was not consistently available in the retrospective records, time‐to‐event analyses, including Kaplan–Meier survival curves and Cox proportional hazards regression, could not be reliably performed. Therefore, 90‐day mortality was analyzed as a binary outcome, defined as alive or dead within 90 days after the index PCI.

### 2.5 Data Collection

Data were collected retrospectively from the medical records of patients treated at An‐Najah National University Hospital. A structured Google Form was used as a standardized data‐extraction tool to collect the required demographic, clinical, laboratory, procedural, and outcome variables from the patients' records. The form was designed with predefined fields to minimize variability in data entry and to ensure that the same variables were collected for all included patients. After data extraction, the dataset was reviewed for completeness, consistency, duplicate entries, and out‐of‐range values. Incomplete records were excluded according to the study criteria, and any unclear entries were rechecked against the original medical records before final analysis. The final cleaned dataset was then exported for statistical analysis.

### 2.6 Statistical Analysis

All statistical analyses were performed using IBM SPSS Statistics. Continuous variables were summarized as median and interquartile range (IQR), while categorical variables were presented as frequencies and percentages. The platelet‐to‐lymphocyte ratio (PLR) was evaluated separately before and after PCI. Receiver operating characteristic (ROC) curve analysis was performed to assess the discriminatory ability of pre‐PCI and post‐PCI PLR for the study outcomes. The optimal cutoff values were determined using Youden's index. Based on the outcome‐specific ROC/Youden approach, pre‐PCI PLR was dichotomized at 21.68, derived from the ROC analysis for the composite in‐hospital clinical event, and post‐PCI PLR was dichotomized at 14.85, derived from the ROC analysis for heart failure. Patients were then categorized into low and high PLR groups according to these cutoffs: pre‐PCI PLR < 21.68 versus ≥ 21.68, and post‐PCI PLR < 14.85 versus ≥ 14.85. Between‐group comparisons were performed. Continuous variables were compared using the Mann–Whitney U test/Wilcoxon rank‐sum test, while categorical variables were compared using the chi‐square test or Fisher's exact test, as appropriate.

Multivariable logistic regression analysis was used to evaluate whether PLR independently predicted clinical outcomes. PLR was entered into the models in two formats: first as a continuous variable, reported per 10‐unit increase, and second as a dichotomous variable using the ROC‐derived cutoff. The evaluated outcomes included recorded adverse events, derived composite in‐hospital clinical events, heart failure, and 90‐day mortality. Models were adjusted for age, sex, smoking status, hypertension, diabetes mellitus, renal failure on dialysis, and preprocedural shock. Body mass index and left ventricular ejection fraction were not included in the main adjusted models because of high missingness. Results were reported as adjusted odds ratios (aORs) with 95% confidence intervals (CIs). A two‐sided *p*‐value < 0.05 was considered statistically significant. Models with sparse events or separation were considered unstable or not estimable and were reported accordingly.

### 2.7 Ethical Considerations

Our study was approved by the research committee of An‐Najah National University and An‐Najah National University Hospital IRB committee (reference #: Med.Aug. 2025/47). Before initiating the study, access and use of the participants' clinical information were authorized by the IRB. The study was done under their ethical guidelines. This study was a retrospective chart review based solely on previously recorded medical data. Informed consent was waived by the Institutional Review Board due to the retrospective design and the use of existing medical records with no direct participant contact (and no additional interventions). Each participant was assigned a unique identification number to protect their privacy; only the researchers and the research supervisors have access to the data.

## 3 Results

### 3.1 Baseline and Demographic Characteristics by Pre‐PCI PLR

The patients included in this study were categorized according to their preprocedural platelet‐to‐lymphocyte ratio (PLR), as follows: 202 patients (74.3%) with PLR < 21.68, and 70 patients (25.7%) with PLR ≥ 21.68. The comparison of demographic and clinical characteristics showed that there was no significant difference between groups regarding age (59 (51.8–66) vs. 60 (54.2–65) years; *p* = 0.425), BMI (28.6 (26.4–31.5) vs. 26.1 (24.3–30.1); *p* = 0.227), and the distribution of sex (85.6% vs. 84.3% males; *p* = 0.794). Cardiovascular risk factors such as current smoking habits (55.9% vs. 54.3%; *p* = 0.81), hypertension (49.0% vs. 56.5%; *p* = 0.281), diabetes mellitus (38.6% vs. 42.6%; *p* = 0.554), and dyslipidemia (4.0% vs. 5.8%; *p* = 0.508) were comparable. There was also no difference concerning the history of prior myocardial infarction, peripheral vascular disease, PCI, CABG, dialysis‐dependent renal failure, arrhythmia, heart failure, and NYHA classification (all *p* > 0.1). History of prior anemia was noted to have a borderline difference between groups (1.5% vs. 5.7%; *p* = 0.075). LVEF tended to be decreased among patients with PLR ≥ 21.68 (45 (35–55) vs. 50 (40–55); *p* = 0.051). Moreover, the history of cerebrovascular disease was more common among patients with PLR ≥ 21.68 (7.2% vs. 2.0%; *p* = 0.05), with borderline statistical signifiance. Preprocedural plasma troponin levels in higher PLR patients were also significantly increased compared to those in lower PLR subjects (99 [17–1449] vs. 34.1 [9.69–154.8] ng/L; *p* = 0.003). Medication profiles, including antiplatelet agents, anticoagulants, beta‐blockers, ACE inhibitors, and statins, showed no significant differences between groups (all *p* > 0.05) (Table 1).

**TABLE 1:** Baseline clinical and demographic characteristics stratified by preprocedural platelet‐to‐lymphocyte ratio (PLR) (< 21.68 vs. ≥ 21.68).

Characteristic	pre‐PCI PLR < 21.68	pre‐PCI PLR ≥ 21.68	*p* value
	*n* = 202	*n* = 70	
Demographic Characteristics			
Age, median (IQR)	59 (51.8, 66)	60 (54.2, 65)	0.425
Sex			0.794
Male	172 (85.6%)	59 (84.3%)	
Female	29 (14.4%)	11 (15.7%)	
BMI, median (IQR)	28.6 (26.4, 31.5)	26.1 (24.3, 30.1)	0.227
Cardiovascular risk factors			
Smoking	113 (55.9%)	38 (54.3%)	0.81
Hypertension	99 (49.0%)	39 (56.5%)	0.281
Diabetes mellitus	76 (38.6%)	29 (42.6%)	0.554
Dyslipidemia	8 (4.0%)	4 (5.8%)	0.508
Previous medical history			
Previous MI			0.637
0	174 (86.1%)	62 (88.6%)	
1	19 (9.4%)	7 (10.0%)	
2	4 (2.0%)	0 (0.0%)	
≥ 3	5 (2.5%)	1 (1.4%)	
Previous peripheral vascular disease	3 (1.5%)	1 (1.4%)	> 0.9
Previous cerebrovascular disease	4 (2.0%)	5 (7.2%)	0.050
Previous PCI			0.793
0	161 (79.7%)	57 (82.6%)	
1	30 (14.9%)	10 (14.5%)	
2	3 (1.5%)	1 (1.4%)	
≥ 3	8 (4.0%)	1 (1.4%)	
Previous CABG	7 (3.5%)	0 (0.0%)	0.196
Previous renal failure on dialysis	15 (7.4%)	5 (7.1%)	> 0.9
Previous diagnosed arrhythmia	4 (2.0%)	1 (1.4%)	> 0.9
Previous history of heart failure	9 (4.6%)	2 (2.9%)	0.733
Previous NYHA status			0.131
I	21 (10.4%)	13 (18.8%)	
II	150 (74.6%)	50 (72.5%)	
III	29 (14.4%)	5 (7.2%)	
IV	1 (0.5%)	1 (1.4%)	
History of anemia	3 (1.5%)	4 (5.7%)	0.075
Clinical and laboratory characteristics			
Preprocedural shock	1 (0.5%)	2 (2.9%)	0.166
Preprocedural creatinine, median (IQR)	0.9 (0.8, 1.1)	0.98 (0.8, 1.28)	0.113
Preprocedural troponin, median (IQR)	34.1 (9.69, 154.8)	99 (17, 1449)	0.003*
Preprocedural LVEF, median (IQR)	50 (40, 55)	45 (35, 55)	0.051
Baseline medications			
Aspirin	58 (28.9%)	19 (27.9%)	0.885
Clopidogrel	19 (9.5%)	10 (14.7%)	0.227
Warfarin	2 (1.0%)	0 (0.0%)	> 0.9
Other anticoagulant	3 (1.5%)	0 (0.0%)	0.573
Beta blocker	41 (20.4%)	15 (21.7%)	0.813
Nitrates	0 (0.0%)	1 (1.5%)	0.252
CCB	30 (15.0%)	14 (20.3%)	0.306
ACE inhibitor	16 (7.9%)	1 (1.4%)	0.081
ARB	47 (23.3%)	12 (17.4%)	0.307
Statin	42 (20.8%)	15 (22.4%)	0.782

*Note*: Data are presented as median (interquartile range) or *n* (%). For binary variables, only the “Yes” category is presented. Percentages were calculated using the available data for each variable; therefore, denominators may vary because of missing values. Continuous variables were compared using the Mann–Whitney *U* test, and categorical variables were compared using the chi‐square test or Fisher's exact test, as appropriate. *p < 0.05.

### 3.2 Procedural and Angiographic Characteristics by Pre‐PCI PLR

For the entire population, there was no difference between total ischemic time (220 [115–450] vs. 292.5 [115–830] minutes; *p* = 0.259), door‐to‐balloon time of < 90 min in the first run (13.0% vs. 16.2%; *p* = 0.512), access route (radial access 88.1% vs. 85.5%; *p* = 0.629), and the proportion of successfully recanalized vessels (88.3% vs. 88.1%; *p* > 0.9). Interestingly, the time from initial ECG to transfer was significantly shorter in patients with higher PLR values (10 [5–10] vs. 10 [10–15] minutes; *p* = 0.046). The use of IABP was significantly higher in patients with PLR ≥ 21.68 (60.0% vs. 42.3%; *p* = 0.011). No significant differences were observed between the groups in PCI technique, stent type, residual distal stenosis > 50%, or the use of heparin, bivalirudin, and GP IIb/IIIa inhibitors (Table 2).

**TABLE 2:** Procedural and angiographic characteristics stratified by preprocedural platelet‐to‐lymphocyte ratio (PLR) (< 21.68 vs. ≥ 21.68).

Characteristic	pre‐PCI PLR < 21.68	pre‐PCI PLR ≥ 21.68	*p* value
	*n* = 202	*n* = 70	
Treatment time intervals			
Symptoms‐to‐FMC time, min, median (IQR)	180 (60, 420)	300 (120, 840)	0.156
FMC‐to‐first‐door time, min, median (IQR)	0 (0, 0)	0 (0, 0)	0.765
First ECG‐to‐transfer time, min, median (IQR)	10 (10, 15)	10 (5, 10)	0.046*
First hospital‐to‐transport time, min, median (IQR)	20 (10, 20)	20 (10, 20)	0.857
Ambulance transport time, min, median (IQR)	15 (5, 30)	15 (5, 30)	0.688
Tertiary arrival‐to‐balloon time, min, median (IQR)	5 (5, 7.5)	5 (5, 10)	0.303
Total ischemic time, min, median (IQR)	220 (115, 450)	292.5 (115, 830)	0.259
First door‐to‐balloon time < 90 min	26 (13.0%)	11 (16.2%)	0.512
First door‐to‐balloon time < 120 min	49 (24.4%)	19 (27.5%)	0.602
Procedural and angiographic characteristics			
Route of entry			0.629
Radial	177 (88.1%)	59 (85.5%)	
Femoral	19 (9.5%)	9 (13.0%)	
Brachial	5 (2.5%)	1 (1.4%)	
Intra‐aortic balloon pump use	85 (42.3%)	42 (60.0%)	0.011*
Successful recanalization	174 (88.3%)	59 (88.1%)	> 0.9
Number of attempts			0.209
0	3 (4.8%)	0 (0.0%)	
1	54 (85.7%)	32 (97.0%)	
2	6 (9.5%)	1 (3.0%)	
3	0 (0.0%)	0 (0.0%)	
PCI technique/device			0.756
None	28 (14.5%)	12 (17.1%)	
Balloon	9 (4.7%)	3 (4.3%)	
Stent	22 (11.4%)	5 (7.1%)	
Rotablation	0 (0.0%)	0 (0.0%)	
Mixed	134 (69.4%)	50 (71.4%)	
Stent type			> 0.9
DES	157 (99.4%)	55 (100.0%)	
DCB	1 (0.6%)	0 (0.0%)	
Residual distal stenosis > 50%	63 (31.7%)	28 (40.0%)	0.205
Procedural medications			
Heparin	192 (98.0%)	70 (100.0%)	0.576
Bivalirudin	1 (0.5%)	0 (0.0%)	> 0.9
GP IIb/IIIa inhibitor	62 (32.1%)	28 (41.2%)	0.177

*Note:* Data are presented as median (interquartile range) or *n* (%). For binary variables, only the “Yes” category is presented. Percentages were calculated using the available data for each variable; therefore, denominators may vary because of missing values. Continuous variables were compared using the Mann–Whitney *U* test, and categorical variables were compared using the chi‐square test or Fisher's exact test, as appropriate. *p < 0.05.

### 3.3 Post‐Procedural Laboratory Results, In‐Hospital Events, and 90‐Day Outcomes by Pre‐PCI PLR

The creatinine levels after the procedure were significantly higher for patients who had a PLR level ≥ 21.68 than those whose PLR levels were less than that at 0.91 [0.80–1.40] mg/dL compared to 0.90 [0.73–1.10] mg/dL, *p* = 0.043. Postprocedural troponin was also significantly higher in the PLR ≥ 21.68 group at 2252 [111–5887] ng/L compared to those in the less than 21.68 group at 735 [40.4–2355.5] ng/L, *p* = 0.013. Postprocedural hemoglobin was not statistically different between the two groups at 14.0 [13.0–15.0] g/dL and 13.8 [11.1–15.2] g/dL, respectively. Regarding in‐hospital events, there was a statistically significant relationship when it came to the occurrence of life‐threatening arrhythmia in the higher PLR value at 5.9% compared to 1.0%, *p* = 0.039, as well as the requirement of circulatory support for patients in the PLR ≥ 21.68 group at 18.8% compared to 9.5%, *p* = 0.040. The composite in‐hospital clinical event rates were higher at 42.9% compared to 20.8%, *p* < 0.001. However, intraprocedural complications (*p* = 0.101), acute congestive heart failure (1.5% vs. 0.5%; *p* = 0.445), mechanical ventilation (10.1% vs. 4.0%; *p* = 0.071), cardiogenic shock (10.0% vs. 4.5%; *p* = 0.136), heart failure (11.4% vs. 5.0%; *p* = 0.09), recorded adverse events (*p* > 0.9), pericardial tamponade (*p* = 0.259), CVA/stroke (*p* = 0.273), and need for dialysis (*p* = 0.542) did not achieve statistical significance. Hospital length of stay was significantly longer in the PLR ≥ 21.68 group (3 [2–4] vs. 2 [1–3] days; *p* < 0.001), and postprocedural LVEF was significantly lower (45 [31.2–55] vs. 50 [40–55]; *p* = 0.037). No intraprocedural mortality was recorded in either group. Death within 90 days (4.5% vs. 3.5%; *p* = 0.716), CABG during admission (*p* = 0.375), bleeding events (*p* = 0.445), vascular complications (*p* = 0.063), and RBC transfusion (*p* = 0.053) remained comparable (Table 3).

**TABLE 3:** Post‐procedural laboratory values, in‐hospital complications, and 90‐day outcomes stratified by preprocedural platelet‐to‐lymphocyte ratio (PLR) (< 21.68 vs. ≥ 21.68).

Characteristic	pre‐PCI PLR < 21.68	pre‐PCI PLR ≥ 21.68	*p* value
	*n* = 202	*n* = 70	
Postprocedural laboratory findings			
Postprocedural creatinine, median (IQR)	0.9 (0.73, 1.1)	0.91 (0.8, 1.4)	0.043*
Postprocedural hemoglobin, median (IQR)	14 (13, 15)	13.8 (11.1, 15.2)	0.16
Postprocedural troponin, median (IQR)	735 (40.4, 2355.5)	2252 (111, 5887)	0.013*
Intraprocedural events			
Intraprocedural complications	3 (5.0%)	5 (18.5%)	0.101
Acute CHF	1 (0.5%)	1 (1.5%)	0.445
Life‐threatening arrhythmia	2 (1.0%)	4 (5.9%)	0.039*
Mechanical ventilation	8 (4.0%)	7 (10.1%)	0.071
Circulatory support	19 (9.5%)	13 (18.8%)	0.04*
Intraprocedural mortality	0 (0.0%)	0 (0.0%)	—
In‐hospital outcomes			
Derived composite in‐hospital clinical event	42 (20.8%)	30 (42.9%)	< 0.001*
Cardiogenic shock	9 (4.5%)	7 (10.0%)	0.136
Heart failure	10 (5.0%)	8 (11.4%)	0.09
Pericardial tamponade	0 (0.0%)	1 (1.4%)	0.259
CVA/stroke	2 (1.0%)	2 (2.9%)	0.273
Postprocedural dialysis	10 (5.0%)	5 (7.2%)	0.542
Hospital stay, days, median (IQR)	2 (1, 3)	3 (2, 4)	< 0.001*
Postprocedural LVEF, median (IQR)	50 (40, 55)	45 (31.2, 55)	0.037*
CABG during admission	4 (2.0%)	3 (4.4%)	0.375
Bleeding event within 72 h	1 (0.5%)	1 (1.4%)	0.445
Vascular complication requiring treatment	0 (0.0%)	2 (2.9%)	0.063
RBC/whole‐blood transfusion	1 (0.5%)	3 (4.3%)	0.053
Follow‐up outcome			
90‐day mortality	7 (3.5%)	3 (4.5%)	0.716

*Note:* Data are presented as median (interquartile range) or *n* (%). For binary variables, only the “Yes” category is presented. Percentages were calculated using the available data for each variable; therefore, denominators may vary because of missing values. Continuous variables were compared using the Mann–Whitney *U* test, and categorical variables were compared using the chi‐square test or Fisher's exact test, as appropriate. *p < 0.05.

### 3.4 Baseline and Demographic Characteristics by Post‐PCI PLR

According to stratification based on postprocedural PLR, there were 163 patients (65.5%) with PLR < 14.85 and 86 patients (34.5%) with PLR ≥ 14.85. Patients with higher postprocedural PLR were significantly older (median age 60 [54.2–70] vs. 58 [51–64] years; *p* = 0.026). Smoking was significantly less prevalent in the higher PLR group (46.5% vs. 65.0%; *p* = 0.005). Other cardiovascular risk factors, such as arterial hypertension (51.8% vs. 49.1%; *p* = 0.688), diabetes mellitus (42.9% vs. 38.0%; *p* = 0.46), and dyslipidemia (4.7% vs. 4.3%; *p* > 0.9) were equally present in both groups. The proportion of prior arrhythmia was significantly different, with a greater prevalence of arrhythmia among the group with PLR ≥ 14.85 (4.7% vs. 0%; *p* = 0.014). Preprocedural LVEF was significantly lower in patients with higher PLR (43.5 [33.5–50] vs. 50 [40–60]; *p* < 0.001). Preprocedural troponin values were significantly higher (75 [15–1095] vs. 31.2 [6.57–153.7] ng/L; *p* = 0.004). The body mass index (BMI) was numerically lower in the ≥ 14.85 group, but this was not statistically significant (25.9 [24.3–29.4] vs. 28.5 [26.5–32.2]; *p* = 0.083). Histories of previous MI, peripheral vascular disease, cerebrovascular disease, previous PCI, CABG, renal failure on dialysis, heart failure, NYHA classification, anemia (*p* = 0.238), and preprocedural shock were all comparable between groups (all *p* > 0.1). Medication profiles, including all antiplatelet agents, anticoagulants, beta‐blockers, and cardiovascular drugs, showed no significant differences (all *p* > 0.3) (Table 4).

**TABLE 4:** Baseline clinical and demographic characteristics stratified by postprocedural platelet‐to‐lymphocyte ratio (PLR) (< 14.85 vs. ≥ 14.85).

Characteristic	post‐PCI PLR < 14.85	post‐PCI PLR ≥ 14.85	*p* value
	*n* = 163	*n* = 86	
Demographic Characteristics			
Age, median (IQR)	58 (51, 64)	60 (54.2, 70)	0.026*
Sex			0.541
Male	142 (87.7%)	73 (84.9%)	
Female	20 (12.3%)	13 (15.1%)	
BMI, median (IQR)	28.5 (26.5, 32.2)	25.9 (24.3, 29.4)	0.083
Cardiovascular risk factors			
Smoking	106 (65.0%)	40 (46.5%)	0.005*
Hypertension	80 (49.1%)	44 (51.8%)	0.688
Diabetes mellitus	60 (38.0%)	36 (42.9%)	0.46
Dyslipidemia	7 (4.3%)	4 (4.7%)	> 0.9
Previous medical history			
Previous MI			0.302
0	143 (87.7%)	72 (83.7%)	
1	12 (7.4%)	12 (14.0%)	
2	3 (1.8%)	1 (1.2%)	
≥ 3	5 (3.1%)	1 (1.2%)	
Previous peripheral vascular disease	4 (2.5%)	1 (1.2%)	0.662
Previous cerebrovascular disease	6 (3.7%)	4 (4.7%)	0.739
Previous PCI			0.56
0	130 (79.8%)	69 (81.2%)	
1	23 (14.1%)	14 (16.5%)	
2	3 (1.8%)	1 (1.2%)	
≥ 3	7 (4.3%)	1 (1.2%)	
Previous CABG	5 (3.1%)	1 (1.2%)	0.668
Previous renal failure on dialysis	9 (5.5%)	7 (8.1%)	0.423
Previous diagnosed arrhythmia	0 (0.0%)	4 (4.7%)	0.014*
Previous history of heart failure	5 (3.2%)	6 (7.1%)	0.197
Previous NYHA status			> 0.9
I	24 (14.8%)	11 (12.8%)	
II	116 (71.6%)	63 (73.3%)	
III	21 (13.0%)	11 (12.8%)	
IV	1 (0.6%)	1 (1.2%)	
History of anemia	3 (1.8%)	4 (4.7%)	0.238
Clinical and laboratory characteristics			
Preprocedural shock	0 (0.0%)	2 (2.3%)	0.119
Preprocedural creatinine, median (IQR)	0.93 (0.8, 1.1)	0.9 (0.8, 1.2)	0.453
Preprocedural troponin, median (IQR)	31.2 (6.57, 153.7)	75 (15, 1095)	0.004*
Preprocedural LVEF, median (IQR)	50 (40, 60)	43.5 (33.5, 50)	< 0.001*
Baseline medications			
Aspirin	47 (29.0%)	28 (33.3%)	0.485
Clopidogrel	19 (11.7%)	10 (11.9%)	> 0.9
Warfarin	2 (1.2%)	0 (0.0%)	0.546
Other anticoagulant	1 (0.6%)	2 (2.4%)	0.273
Beta blocker	34 (21.0%)	18 (21.2%)	> 0.9
Nitrates	0 (0.0%)	1 (1.2%)	0.34
CCB	24 (14.9%)	16 (18.8%)	0.429
ACE inhibitor	12 (7.4%)	4 (4.7%)	0.419
ARB	36 (22.2%)	18 (21.2%)	0.85
Statin	34 (21.1%)	21 (25.0%)	0.489

*Note:* Data are presented as median (interquartile range) or *n* (%). For binary variables, only the “Yes” category is presented. Percentages were calculated using the available data for each variable; therefore, denominators may vary because of missing values. Continuous variables were compared using the Mann–Whitney *U* test, and categorical variables were compared using the chi‐square test or Fisher's exact test, as appropriate. *p < 0.05.

### 3.5 Procedural and Angiographic Characteristics by Post‐PCI PLR

The procedural and angiographic features were similar for the patients with PLR < 14.85 and PLR ≥ 14.85 at the postprocedural stage. There were no statistically significant differences in total ischemic time (200 [115–440] vs. 255 [115–840]; *p* = 0.201), door to balloon times < 90 min (15.6% vs. 14.1%; *p* = 0.754), and access method (radial artery in 88.9% vs. 83.5%; *p* = 0.461). No significant differences were detected regarding the use of intra‐aortic balloon pump (49.4% vs. 51.2%; *p* = 0.79), successful recanalization rate (89.4% vs. 91.7%; *p* = 0.578), the spectrum of PCI methods (*p* = 0.528), and the stent type (*p* > 0.9). Residual distal stenosis over 50% was observed more often in the patients with PLR ≥ 14.85 group (43.0% vs. 30.1%; *p* = 0.041). Anticoagulation strategies, including heparin use (98.8% vs. 97.6%; *p* = 0.609), bivalirudin utilization (*p* = 0.546), and GP IIb/IIIa inhibitor administration (35.3% in both groups; *p* > 0.9), were comparable (Table 5).

**TABLE 5:** Procedural and angiographic characteristics stratified by postprocedural platelet‐to‐lymphocyte ratio (PLR) (< 14.85 vs. ≥ 14.85).

Characteristic	post‐PCI PLR < 14.85	post‐PCI PLR ≥ 14.85	*p* value
	*n* = 163	*n* = 86	
Treatment time intervals			
Symptoms‐to‐FMC time, min, median (IQR)	180 (60, 360)	240 (97.5, 1440)	0.065
FMC‐to‐first‐door time, min, median (IQR)	0 (0, 0)	0 (0, 0)	0.147
First ECG‐to‐transfer time, min, median (IQR)	10 (5, 15)	10 (5, 10)	0.299
First hospital‐to‐transport time, min, median (IQR)	20 (10, 20)	20 (10, 20)	0.557
Ambulance transport time, min, median (IQR)	15 (5, 30)	10 (5, 30)	> 0.9
Tertiary arrival‐to‐balloon time, min, median (IQR)	5 (5, 10)	5 (5)	0.526
Total ischemic time, min, median (IQR)	200 (115, 440)	255 (115, 840)	0.201
First door‐to‐balloon time < 90 min	25 (15.6%)	12 (14.1%)	0.754
First door‐to‐balloon time < 120 min	45 (27.8%)	22 (25.9%)	0.75
Procedural and angiographic characteristics			
Route of entry			0.461
Radial	144 (88.9%)	71 (83.5%)	
Femoral	15 (9.3%)	11 (12.9%)	
Brachial	3 (1.9%)	3 (3.5%)	
Intra‐aortic balloon pump use	80 (49.4%)	44 (51.2%)	0.79
Successful recanalization	144 (89.4%)	77 (91.7%)	0.578
Number of attempts			0.078
0	0 (0.0%)	2 (6.1%)	
1	58 (90.6%)	30 (90.9%)	
2	6 (9.4%)	1 (3.0%)	
3	0 (0.0%)	0 (0.0%)	
PCI technique/device			0.528
None	23 (14.3%)	10 (12.2%)	
Balloon	7 (4.3%)	4 (4.9%)	
Stent	21 (13.0%)	6 (7.3%)	
Rotablation	0 (0.0%)	0 (0.0%)	
Mixed	110 (68.3%)	62 (75.6%)	
Stent type			> 0.9
DES	129 (99.2%)	71 (100.0%)	
DCB	1 (0.8%)	0 (0.0%)	
Residual distal stenosis > 50%	49 (30.1%)	37 (43.0%)	0.041*
Procedural medications			
Heparin	159 (98.8%)	82 (97.6%)	0.609
Bivalirudin	2 (1.2%)	0 (0.0%)	0.546
GP IIb/IIIa inhibitor	55 (35.3%)	28 (35.3%)	> 0.9

*Note:* Data are presented as median (interquartile range) or *n* (%). For binary variables, only the “Yes” category is presented. Percentages were calculated using the available data for each variable; therefore, denominators may vary because of missing values. Continuous variables were compared using the Mann–Whitney *U* test, and categorical variables were compared using the chi‐square test or Fisher's exact test, as appropriate. *p < 0.05.

### 3.6 Post‐Procedural Laboratory Results, In‐Hospital Events, and 90‐Day Outcomes by Post‐PCI PLR

Postprocedural levels of creatinine (0.95 [0.78–1.36] vs. 0.88 [0.74–1.04] mg/dL; *p* = 0.041) and troponin (1927 [72.5–5308.8] vs. 447.5 [35.8–1902.5] ng/L; *p* = 0.003) in the PLR ≥ 14.85 cohort were significantly higher compared to the respective postprocedural levels in the PLR < 14.85 group, while postprocedural levels of hemoglobin in both groups did not significantly differ (13.9 [12.3–15.0] vs. 14.1 [12.7–15.0] g/dL; *p* = 0.465). In‐hospital outcomes demonstrated significantly higher rates of mechanical ventilation (11.8% vs. 2.5%; *p* = 0.007), need for circulatory support (21.2% vs. 8.8%; *p* = 0.006), cardiogenic shock (12.8% vs. 1.9%; *p* < 0.001), and heart failure (12.8% vs. 4.3%; *p* = 0.014) in the high PLR group. The composite in‐hospital clinical event rate was also significantly higher in the high‐PLR group (38.4% vs. 20.9%; *p* = 0.003). Patients with elevated post‐PCI PLR also had a longer hospital stay (2 [2–4] vs. 2 [1–3] days; *p* = 0.001), and a lower postprocedural LVEF (40 [30–55] vs. 50 [43–60]; *p* < 0.001). Within the next 90‐day period, the risk of death was significantly higher in the group with high postprocedural PLR (7.1% vs. 0.6%; *p* = 0.007). On the contrary, no significant differences were observed between the groups concerning acute congestive heart failure (CHF) (*p* > 0.9), intraprocedural complications (*p* > 0.9), life‐threatening arrhythmia (3.5% vs. 2.5%; *p* = 0.698), recorded adverse events (*p* = 0.116), pericardial tamponade (*p* > 0.9), CVA/stroke (*p* = 0.275), need for dialysis (*p* = 0.115), CABG during admission (*p* = 0.41), bleeding events within 72 h (*p* = 0.117), vascular complications (*p* = 0.118), and RBC transfusion (*p* = 0.119). No intraprocedural mortality was recorded in either group (Table 6).

**TABLE 6:** Postprocedural laboratory values, in‐hospital complications, and 90‐day outcomes stratified by postprocedural platelet‐to‐lymphocyte ratio (PLR) (< 14.85 vs. ≥ 14.85).

Characteristic	post‐PCI PLR < 14.85	post‐PCI PLR ≥ 14.85	*p* value
	*n* = 163	*n* = 86	
Postprocedural laboratory findings			
Postprocedural creatinine, median (IQR)	0.88 (0.74, 1.04)	0.95 (0.78, 1.36)	0.041*
Postprocedural hemoglobin, median (IQR)	14.1 (12.7, 15)	13.9 (12.3, 15)	0.465
Postprocedural troponin, median (IQR)	447.5 (35.8, 1902.5)	1927 (72.5, 5308.8)	0.003*
Intraprocedural events			
Intraprocedural complications	6 (10.3%)	2 (8.0%)	> 0.9
Acute CHF	1 (0.6%)	1 (1.2%)	> 0.9
Life‐threatening arrhythmia	4 (2.5%)	3 (3.5%)	0.698
Mechanical ventilation	4 (2.5%)	10 (11.8%)	0.007*
Circulatory support	14 (8.8%)	18 (21.2%)	0.006*
Intraprocedural mortality	0 (0.0%)	0 (0.0%)	—
In‐hospital outcomes			
Derived composite in‐hospital clinical event	34 (20.9%)	33 (38.4%)	0.003*
Cardiogenic shock	3 (1.9%)	11 (12.8%)	< 0.001*
Heart failure	7 (4.3%)	11 (12.8%)	0.014*
Pericardial tamponade	1 (0.6%)	0 (0.0%)	> 0.9
CVA/stroke	1 (0.6%)	2 (2.3%)	0.275
Postprocedural dialysis	5 (3.1%)	7 (8.2%)	0.115
Hospital stay, days, median (IQR)	2 (1, 3)	2 (2, 4)	0.001*
Postprocedural LVEF, median (IQR)	50 (43, 60)	40 (30, 55)	< 0.001*
CABG during admission	3 (1.9%)	3 (3.6%)	0.41
Bleeding event within 72 h	0 (0.0%)	2 (2.4%)	0.117
Vascular complication requiring treatment	0 (0.0%)	2 (2.4%)	0.118
RBC/whole‐blood transfusion	1 (0.6%)	3 (3.5%)	0.119
Follow‐up outcome			
90‐day mortality	1 (0.6%)	6 (7.1%)	0.007*

*Note:* Data are presented as median (interquartile range) or *n* (%). For binary variables, only the “Yes” category is presented. Percentages were calculated using the available data for each variable; therefore, denominators may vary because of missing values. Continuous variables were compared using the Mann–Whitney *U* test, and categorical variables were compared using the chi‐square test or Fisher's exact test, as appropriate. *p < 0.05.

### 3.7 Multivariable Logistic Regression Analysis

After controlling for age, sex, smoking, hypertension, diabetes mellitus, end‐stage renal disease needing dialysis, and preprocedural shock, multivariable logistic regression analysis confirmed the independent association of PLR for various clinical endpoints. After considering PLR as a continuous variable, each increase of 10 units in PLR before PCI was independently associated with a significantly increased chance of the derived in‐hospital clinical endpoint (odds ratio [OR] 1.41, 95% CI 1.13–1.74; *p* = 0.002). On the other hand, PLR was not significantly associated with adverse events (OR 1.20; *p* = 0.535), heart failure (OR 1.03; *p* = 0.864), or 90‐day mortality (OR 1.23; *p* = 0.367). Using the cutoff of PLR (≥ 21.68) derived from ROC analysis before PCI, PLR still proved to be an independent predictor of the composite in‐hospital event (OR 2.90, 95% CI 1.55–5.44; *p* < 0.001). However, PLR was not significantly associated with heart failure (OR 2.49; *p* = 0.069) and 90‐day mortality (OR 0.99; *p* > 0.9).

Post‐PCI PLR demonstrated broader independent associations with the evaluated outcomes. Every increase of 10 units of post‐PCI PLR was independently associated with the composite of in‐hospital clinical events (OR 1.65, 95% CI 1.22–2.24; *p* = 0.001), heart failure (OR 1.58, 95% CI 1.07–2.31; *p* = 0.02) and 90‐day mortality (OR 2.46, 95% CI 1.33–4.54; *p* = 0.004). Even using the ROC‐based cutpoint, a PLR value ≥ 14.85 remained independently associated with the composite of in‐hospital events (OR 2.26, 95% CI 1.21–4.20; *p* = 0.01), heart failure (OR 3.68, 95% CI 1.32–10.25; *p* = 0.013), and 90‐day mortality (OR 10.55, 95% CI 1.14–98.15; *p* = 0.038). Logistic regression models for recorded adverse events using post‐PCI PLR were not estimable due to sparse events or complete separation (Table 7).

**TABLE 7:** Adjusted associations of preprocedural and postprocedural PLR with in‐hospital clinical outcomes and 90‐day mortality.

Exposure	Predictor format	Outcome	Events/N	Adjusted OR (95% CI)	*p* value
**Pre‐PCI PLR**	Continuous per 10‐unit increase	Recorded any adverse event	9/241	1.20 (0.67–2.14)	0.535
**Pre‐PCI PLR**	Continuous per 10‐unit increase	Derived composite in‐hospital clinical event	70/260	1.41 (1.13–1.74)	0.002*
**Pre‐PCI PLR**	Continuous per 10‐unit increase	Heart failure	18/259	1.03 (0.71–1.50)	0.864
**Pre‐PCI PLR**	Continuous per 10‐unit increase	90‐day mortality	10/255	1.23 (0.78–1.93)	0.367
**Pre‐PCI PLR**	ROC cutoff ≥ 21.68 versus < 21.68	Recorded any adverse event	9/241	1.16 (0.21–6.38)	0.863
**Pre‐PCI PLR**	ROC cutoff ≥ 21.68 versus < 21.68	Derived composite in‐hospital clinical event	70/260	2.90 (1.55–5.44)	< 0.001*
**Pre‐PCI PLR**	ROC cutoff ≥ 21.68 versus < 21.68	Heart failure	18/259	2.49 (0.93–6.69)	0.069
**Pre‐PCI PLR**	ROC cutoff ≥ 21.68 versus < 21.68	90‐day mortality	10/255	0.99 (0.18–5.27)	> 0.9
**Post‐PCI PLR**	Continuous per 10‐unit increase	Recorded any adverse event	8/220	Not estimable	
**Post‐PCI PLR**	Continuous per 10‐unit increase	Derived composite in‐hospital clinical event	65/238	1.65 (1.22–2.24)	0.001*
**Post‐PCI PLR**	Continuous per 10‐unit increase	Heart failure	18/237	1.58 (1.07–2.31)	0.02*
**Post‐PCI PLR**	Continuous per 10‐unit increase	90‐day mortality	7/235	2.46 (1.33–4.54)	0.004*
**Post‐PCI PLR**	ROC cutoff ≥ 14.85 versus < 14.85	Recorded any adverse event	8/220	Not estimable	
**Post‐PCI PLR**	ROC cutoff ≥ 14.85 versus < 14.85	Derived composite in‐hospital clinical event	65/238	2.26 (1.21–4.20)	0.01*
**Post‐PCI PLR**	ROC cutoff ≥ 14.85 versus < 14.85	Heart failure	18/237	3.68 (1.32–10.25)	0.013*
**Post‐PCI PLR**	ROC cutoff ≥ 14.85 versus < 14.85	90‐day mortality	7/235	10.55 (1.14–98.15)	0.038*

*Note:* Adjusted odds ratios were estimated using multivariable logistic regression adjusted for age, sex, smoking status, hypertension, diabetes mellitus, dialysis‐dependent renal failure, and preprocedural shock. Continuous PLR estimates are reported per 10‐unit increase.

Abbreviations: CI, confidence interval; NE, not estimable because of sparse events or complete separation; OR, odds ratio; PCI, percutaneous coronary intervention; PLR, platelet‐to‐lymphocyte ratio.

## 4 Discussion

This retrospective cohort study assessed the predictive value of platelet‐to‐lymphocyte ratio (PLR) measured before and after PCI in patients presenting with STEMI to a tertiary center in Nablus, Palestine. Although prior similar research has recognized PLR as a prognostic marker in acute coronary syndromes (ACS) [17, 18], our study uniquely contributes by evaluating both pre‐ and postprocedural PLR values and associating them with clinical, angiographic, and 90‐day outcomes while adjusting for important clinical confounders.

Regarding baseline and demographic characteristics, age, sex distribution, and BMI were generally comparable between low and high pre‐PCI PLR groups. The median age was around 60 years, with a strong male predominance, reflecting a younger and more sex‐biased population than most international cohorts. This may have contributed to the overall low incidence of adverse events and muted the predictive value of PLR compared to studies which included older patients with higher baseline risk [19, 20].

None of the other risk factors (smoking, hypertension, diabetes mellitus, hyperlipidemia) showed significant differences between pre‐PCI PLR groups, suggesting that PLR elevations were not driven by these traditional comorbidities. However, in the post‐PCI PLR analysis, patients with higher PLR were older and less frequently smokers, and they had lower baseline LVEF and higher preprocedural troponin levels. These differences suggest that postprocedural PLR may reflect a higher‐risk clinical phenotype, including greater myocardial injury and impaired ventricular function, rather than being only a nonspecific inflammatory marker.

Previous MI, PCI, CABG and Peripheral/Cerebrovascular Disease showed no significant differences between groups in these settings. While higher PLR has previously been linked to extensive coronary disease burden [21, 22], our findings showed comparable cardiac history.

Preprocedural renal failure was not significantly different between PLR groups, although postprocedural creatinine was slightly higher among patients with elevated pre‐PCI and post‐PCI PLR. This aligns with research indicating that inflammation‐related endothelial dysfunction and renal hypoperfusion may contribute to renal impairment in ACS [23]. However, the small absolute differences in creatinine should be interpreted cautiously and may not indicate clinically meaningful renal dysfunction in all patients.

No significant differences were noted in prior heart failure or NYHA functional class in either group, before or after PCI. Interestingly, postprocedural high PLR was associated with a significantly higher incidence of new or worsening heart failure. This association remained significant in multivariable logistic regression, both when post‐PCI PLR was analyzed continuously and when using the ROC‐derived cutoff. These findings suggest post‐PCI PLR may reflect dynamic myocardial stress not captured by baseline NYHA status. This finding is consistent with studies that highlight PLR's role in predicting LV dysfunction [24, 25, 26, 27].

Arrhythmias and anemia showed limited and inconsistent differences across groups. Previously diagnosed arrhythmia was more frequent in the high post‐PCI PLR group, but the overall number of events was small. Therefore, although inflammation may theoretically contribute to electrical instability after STEMI, our findings do not support a strong or independent relationship between PLR and arrhythmic outcomes in this cohort.

Procedural characteristics, including total ischemic time and door‐to‐balloon (D2B) times, were comparable between high and low PLR groups. Notably, the proportion of patients achieving D2B < 90 min remained consistently low across all groups, reflecting the fact that we are a tertiary center receiving STEMI cases from the northern part of the West Bank, due to our unique regional system and political situation. Vascular access site preferences (radial, femoral, brachial) were also similar, although a non‐significant trend toward increased femoral access was observed in higher PLR groups, which has been historically associated with worse outcomes due to higher bleeding risk [28, 29].

The use of an intra‐aortic balloon pump (IABP) was significantly more frequent among patients with elevated pre‐PCI PLR than among those with lower pre‐PCI PLR (60.0% vs. 42.3%; *p* = 0.011). In contrast, IABP use was comparable between the high and low post‐PCI PLR groups (51.2% vs. 49.4%; *p* = 0.790). Interestingly, the incidence of residual distal stenosis > 50% was more frequent in the high post‐PCI PLR group. This may indicate that elevated postprocedural PLR reflects a more complex angiographic or microvascular profile, although causality cannot be inferred. Prior studies have linked higher PLR with thrombus burden and poor angiographic outcomes [30, 31].

Furthermore, use of anticoagulants (heparin, bivalirudin, and glycoprotein IIb/IIIa inhibitors) was evenly distributed across groups, indicating comparable intraprocedural management regardless of inflammatory status as reflected by PLR.

Postprocedural troponin and creatinine were significantly higher in patients with elevated pre‐PCI and post‐PCI PLR, while hemoglobin did not differ significantly. These findings support the biological plausibility of PLR as a marker of more extensive myocardial injury and systemic stress [32, 33]. Pathophysiologically, PLR serves as a biomarker reflecting both thrombotic burden (via elevated platelet count) and systemic inflammatory status (via suppressed lymphocyte count). Inflammation activates platelets and promotes coagulation cascades, contributing to myocardial damage and infarct extension [34, 35, 36], while also triggering endothelial dysfunction and renal hypoperfusion, potentially raising creatinine levels. Additionally, systemic inflammation can suppress erythropoiesis or cause hemodilution, contributing to anemia and altering hemoglobin levels. Despite these theoretical links, our relatively young cohort with a low burden of comorbidities may explain the absence of significant laboratory differences across PLR groups.

Findings of in‐hospital events revealed several trends associated with PLR levels. Elevated preprocedural PLR was associated with higher rates of life‐threatening arrhythmia, need for circulatory support, longer hospital stay, lower postprocedural LVEF, and a higher derived composite in‐hospital clinical event rate. In multivariable logistic regression, pre‐PCI PLR remained independently associated with the derived composite in‐hospital clinical event, both as a continuous variable and using the ROC‐derived cutoff. However, pre‐PCI PLR was not independently associated with recorded adverse events, heart failure, or 90‐day mortality. These findings align with evidence suggesting that an elevated inflammatory response, as indexed by PLR, may predispose patients to complications such as pulmonary edema, respiratory failure, or systemic instability necessitating advanced support [37, 38]. Collectively, these findings suggest that preprocedural PLR may be useful as an early marker of acute in‐hospital instability, but its prognostic value for specific downstream outcomes appears limited.

Postprocedural PLR showed stronger and more consistent prognostic value. Patients with elevated post‐PCI PLR had significantly higher rates of mechanical ventilation, circulatory support, cardiogenic shock, heart failure, longer hospital stay, lower postprocedural LVEF, and 90‐day mortality. In adjusted models, post‐PCI PLR remained independently associated with the derived composite in‐hospital clinical event, heart failure, and 90‐day mortality. This finding may be explained by the different biological information captured by PLR before and after reperfusion. Pre‐PCI PLR primarily reflects the inflammatory and thrombotic state present at hospital admission. However, it is measured before the full effects of myocardial reperfusion, procedural injury, and early postprocedural complications have developed. In contrast, post‐PCI PLR may integrate the baseline inflammatory burden with the subsequent biological response to ischemia–reperfusion and myocardial injury. Reperfusion can promote oxidative stress, endothelial activation, platelet aggregation, leukocyte recruitment, tissue edema, and distal microembolization, all of which may contribute to microvascular obstruction despite restoration of epicardial coronary flow. Persistent platelet activation, together with stress‐related lymphopenia, may therefore increase post‐PCI PLR among patients with greater infarct burden, impaired microvascular perfusion, and more severe systemic stress [11, 13, 19, 34]. Consequently, post‐PCI PLR may better reflect ongoing thrombo‐inflammatory activity and early myocardial damage than a single measurement obtained before PCI. This interpretation is also supported by the higher postprocedural troponin levels, lower LVEF, and greater frequency of residual distal stenosis and hemodynamic complications observed among patients with elevated post‐PCI PLR in our cohort (Tables 5 and 6).

Importantly, the association between post‐PCI PLR and heart failure is one of the most clinically relevant findings of this study. Elevated postprocedural PLR may serve as a practical and inexpensive marker to identify patients at increased risk of early post‐MI heart failure. This is consistent with previous studies reporting a strong relationship between elevated PLR and adverse ventricular remodeling and reduced systolic function in acute coronary syndrome patients [39, 40]. Greater infarct burden and microvascular obstruction may reduce myocardial salvage, impair ventricular recovery, and promote adverse left ventricular remodeling, providing a plausible explanation for the observed association between post‐PCI PLR and heart failure.

In terms of 90‐day outcomes, elevated post‐PCI PLR, but not pre‐PCI PLR, was independently associated with 90‐day mortality in multivariable analysis. However, this finding should be interpreted cautiously because the number of deaths was small, as reflected by the wide confidence interval in the cutoff‐based model. Therefore, while post‐PCI PLR may help identify patients at higher short‐term risk, larger studies are needed to confirm its mortality‐predictive value. Several studies have reported higher mortality associated with elevated PLR [41, 42, 43]. Overall, our results suggest that postprocedural PLR is more informative than preprocedural PLR for risk stratification after primary PCI in STEMI patients.

## 5 Limitations

First, the retrospective design may introduce selection bias, although we attempted to control for this using multivariable adjustment. Second, the single‐center setting at An‐Najah National University Hospital may limit the generalizability of the findings to other regions. Third, our cohort is notably younger than typical STEMI populations, potentially explaining the low observed mortality rate and lack of statistical significance for certain adverse events. In addition, certain factors like infarct size, medication adherence post‐discharge, and detailed long‐term follow‐up data were not captured in the medical records. Also, post‐PCI PLR was calculated from the first available blood count after PCI rather than from samples obtained at a uniform time point; therefore, variation in sampling time and the influence of early complications or treatments may have affected postprocedural PLR values. Additionally, the adjusted mortality models were based on a small number of deaths relative to the number of included covariates and may therefore be overfitted and statistically unstable; these findings should be regarded as exploratory. Finally, the ROC‐derived PLR cutoffs require external validation before they can be applied in routine clinical practice.

## 6 Future Directions

Prospective, multicenter studies are warranted to validate the cutoff values derived in this study. Additionally, studies with larger populations and extended follow‐up beyond 90 days are needed to determine whether PLR‐guided clinical monitoring or interventions can reduce long‐term morbidity and mortality.

## 7 Conclusion

This study highlights the potential utility of postprocedural platelet‐to‐lymphocyte ratio (PLR) as a predictive marker for adverse outcomes following primary PCI in STEMI patients. While preprocedural PLR was independently associated with the composite in‐hospital clinical endpoint, it was not independently associated with heart failure or 90‐day mortality. In contrast, postprocedural PLR showed stronger prognostic value and was independently associated with composite in‐hospital events, heart failure, and 90‐day mortality. Given its simplicity, cost‐effectiveness, and availability from routine blood counts, PLR may serve as a practical adjunctive tool in risk stratification protocols, particularly in resource‐constrained healthcare settings like Palestine. Future multicenter prospective studies with longer follow‐up are essential to validate these findings and explore the mechanistic pathways linking PLR to adverse cardiac remodeling and short‐term mortality.

## Author Contributions

**Anwar Zahran:** conceptualization, writing, review and editing. **Saja Amer:** writing, review and editing. **Mohammad Bdair:** data analysis, writing, review and editing. **Seema Hameedi:** data collection and review. **Roa Badran:** data collection and review. **Mays Safa:** data collection and review. **Abdelfattah M. Dahmas:** writing and review. **Ahmad Mohammad:** writing, review and editing. **Abdul‐rahman Abdel‐karim:** Supervision, writing, review and editing. **Zaher Nazzal:** Supervision, writing, review and editing.

## Funding

The authors have nothing to report.

## Consent

The authors have nothing to report.

## Conflicts of Interest

The authors declare no conflicts of interest.

## Supporting information


Supporting File

## Data Availability

The data sets used and analyzed during the study are available from the corresponding author upon reasonable request.
