# Supplementary material for: The Predictive Value of Platelet‐to‐Lymphocyte Ratio Before and After Percutaneous Coronary Intervention in Patients With Acute ST‐Segment Elevation Myocardial Infarction: A Retrospective Cohort Study in Palestine
Source: Clin Cardiol. 2026 Jul 27;49(7):e70422. doi: 10.1002/clc.70422 (PMC13403153; doi:10.1002/clc.70422)
Supplement: Supplementary file 1 — Supporting File [file CLC-49-e70422-s001.docx]

**Supplementary Table S1.** Study variables and operational definitions.

| **Domain** | **Data type** | **Variable** | **Operational definition** | **Coding / units** |
| --- | --- | --- | --- | --- |
| Exposure/grouping | Continuous and Categorical (binary) | Platelet-to-lymphocyte ratio (PLR) | Calculated by dividing the platelet count by the absolute lymphocyte count, with both values expressed in the same units. | Continuous; categorized as pre-PCI PLR ≥21.68 vs <21.68 and post-PCI PLR ≥14.85 vs <14.85 |
| Baseline demographics | Continuous | Age | Recorded at the time of presentation | Years |
|  | Categorical | Sex | Recorded at the time of presentation | Male / Female |
|  | Continuous | BMI | Recorded at the time of presentation | kg/m² |
| Baseline cardiovascular risk factors | Categorical | Smoking status | Smoking status as documented in the medical record | Yes / No |
|  | Categorical | Hypertension | History documented in the medical record | Yes / No |
|  | Categorical | Diabetes mellitus | History documented in the medical record | Yes / No |
|  | Categorical | Dyslipidemia | History documented in the medical record | Yes / No |
| Past cardiovascular history | Categorical | Previous MI | History of prior myocardial infarction and number of events | 0 / 1 / 2 / ≥3 |
|  | Categorical | Previous peripheral vascular disease | History documented in the medical record | Yes / No |
|  | Categorical | Previous cerebrovascular disease | History documented in the medical record | Yes / No |
|  | Categorical | Previous PCI | History of prior PCI and number of procedures | 0 / 1 / 2 / ≥3 |
|  | Categorical | Previous CABG | History documented in the medical record | Yes / No |
|  | Categorical | Renal failure on dialysis | History documented in the medical record | Yes / No |
|  | Categorical | Prior arrhythmia subtype | Previously diagnosed arrhythmia type | Atrial / Ventricular / Other |
|  | Categorical | History of heart failure | History documented in the medical record | Yes / No |
|  | Ordinal | Prior NYHA class | NYHA functional class documented before the index event | I / II / III / IV |
|  | Continuous | Pre-event ejection fraction | Most recent ejection fraction recorded before the index event | % or proportion (as recorded) |
|  | Categorical | History of anemia | History documented in the medical record | Yes / No |
| Baseline clinical status | Categorical | Preprocedural shock | Presence of shock prior to the procedure | Yes / No |
| Baseline labs | Continuous | Preprocedural creatinine | Pre-procedural creatinine level | mg/dL |
|  | Continuous | Preprocedural troponin | Pre-procedural troponin level | As recorded |
| Home medications (baseline) | Categorical | Aspirin | Medication use prior to hospital arrival (home/prehospital) | Yes / No |
|  | Categorical | Clopidogrel | Medication use prior to hospital arrival (home/prehospital) | Yes / No |
|  | Categorical | Other P2Y12 inhibitors | Medication use prior to hospital arrival (home/prehospital) | Yes / No |
|  | Categorical | Warfarin | Medication use prior to hospital arrival (home/prehospital) | Yes / No |
|  | Categorical | Other anticoagulants | Medication use prior to hospital arrival (home/prehospital) | Yes / No |
|  | Categorical | Digoxin | Medication use prior to hospital arrival (home/prehospital) | Yes / No |
|  | Categorical | Beta blocker | Medication use prior to hospital arrival (home/prehospital) | Yes / No |
|  | Categorical | Nitrates | Medication use prior to hospital arrival (home/prehospital) | Yes / No |
|  | Categorical | Calcium channel blocker | Medication use prior to hospital arrival (home/prehospital) | Yes / No |
|  | Categorical | ACE inhibitors | Medication use prior to hospital arrival (home/prehospital) | Yes / No |
|  | Categorical | ARBs | Medication use prior to hospital arrival (home/prehospital) | Yes / No |
|  | Categorical | Statin | Medication use prior to hospital arrival (home/prehospital) | Yes / No |
| Time intervals (minutes) | Categorical | First-door-to-balloon <90 | Whether first door-to-balloon time met the <90-minute target | Yes / No |
|  | Continuous | Total ischemic time | Time from symptom onset to reperfusion | Minutes |
| Procedural / angiographic | Categorical | Access site | Vascular access route | Radial / Femoral / Brachial |
|  | Ordinal | Number of diseased vessels | Extent of coronary artery disease | 1 / 2 / ≥3 |
|  | Categorical | IABP use | Use of intra-aortic balloon pump | Yes / No |
|  | Categorical | Successful recanalization | Procedural success based on angiographic criteria | Yes / No |
|  | Ordinal | Number of attempts | Number of procedural attempts | 0 / 1 / 2 / ≥3 |
|  | Categorical | PCI technique/devices | PCI strategy/devices used, as recorded | None / Balloon / Stent / Rotablation / Mixed (as recorded) |
|  | Categorical | Residual distal stenosis >50% | Residual distal stenosis after PCI | Yes / No |
| Procedural medications | Categorical | Heparin | Administered during the procedure | Yes / No |
|  | Categorical | Bivalirudin | Administered during the procedure | Yes / No |
|  | Categorical | GP IIb/IIIa inhibitors | Administered during the procedure | Yes / No |
| Post-procedural labs | Continuous | Post-procedural creatinine | Post-procedural creatinine level | mg/dL |
|  | Continuous | Post-procedural hemoglobin | Post-procedural hemoglobin level | g/dL |
|  | Continuous | Post-procedural troponin | Post-procedural troponin level | As recorded |
| Intra-procedural complications | Categorical | Acute CHF | Event occurring during the procedure | Yes / No |
|  | Categorical | Life-threatening arrhythmia | Event occurring during the procedure | Yes / No |
|  | Categorical | Mechanical ventilation | Required during the procedure | Yes / No |
|  | Categorical | Circulatory support | Required during the procedure | Yes / No |
|  | Categorical | Intra-procedural mortality | Death occurring during the procedure | Yes / No |
|  | Categorical | Any intra-procedural complication | Composite indicator for any recorded intra-procedural event | Yes / No |
| In-hospital outcomes (post-procedure) | Categorical | Derived composite in-hospital clinical event | Occurrence of at least one prespecified in-hospital clinical event during the index hospitalization, including acute congestive heart failure, life-threatening arrhythmia, mechanical ventilation, circulatory support, cardiogenic shock, heart failure, cardiac tamponade, CVA/stroke, postprocedural dialysis, intraprocedural mortality, CABG during admission, bleeding within 72 hours, vascular complications requiring treatment, or RBC/whole-blood transfusion | Yes / No |
|  | Categorical | Recorded any adverse event | Indicator of any in-hospital adverse event | Yes / No |
|  | Categorical | Cardiogenic shock | Event occurring after the procedure during the index admission | Yes / No |
|  | Categorical | Heart failure | Event occurring after the procedure during the index admission | Yes / No |
|  | Categorical | Pericardial tamponade | Event occurring after the procedure during the index admission | Yes / No |
|  | Categorical | CVA / stroke | Event occurring after the procedure during the index admission | Yes / No |
|  | Categorical | Dialysis post-procedure | Requirement for dialysis after PCI during the index admission | Yes / No |
|  | Continuous | Length of stay | Duration of hospitalization | Days |
|  | Continuous | Post-procedural EF | Ejection fraction measured after PCI during admission | % or proportion |
|  | Categorical | CABG during admission | CABG performed during the index admission | Yes / No |
|  | Categorical | Bleeding event within 72 hours | Bleeding event within 72 hours after PCI | Yes / No |
|  | Categorical | Vascular complications requiring treatment | Access-site or vascular complication requiring treatment | Yes / No |
|  | Categorical | RBC/whole-blood transfusion | Transfusion during the index admission | Yes / No |
| 90-day outcome | Categorical | Death within 90 days | All-cause death within 90 days | Yes / No |

## Supplementary Table S2. Receiver operating characteristic (ROC) analysis and Youden index derived optimal cutoffs for preprocedural and postprocedural Platelet-to-Lymphocyte Ratio (PLR) for each clinical outcome

| **Exposure** | **Outcome** | **N** | **Events** | **Non-events** | **AUC** | **Youden cutoff** | **Sensitivity** | **Specificity** | **Youden J** | **Selected cutoff for main analyses** |
| --- | --- | --- | --- | --- | --- | --- | --- | --- | --- | --- |
| **Pre-PCI PLR** | Recorded any adverse event | 251 | 9 | 242 | 0.504 | 12.99 | 66.7% | 45.0% | 0.117 |  |
| **Pre-PCI PLR** | Derived composite in-hospital clinical event | 272 | 72 | 200 | 0.613 | 21.68 | 41.7% | 80.0% | 0.217 | Main pre-PCI cutoff used in tables |
| **Pre-PCI PLR** | Heart failure | 271 | 18 | 253 | 0.544 | 21.68 | 44.4% | 75.5% | 0.199 |  |
| **Pre-PCI PLR** | 90-day mortality | 266 | 10 | 256 | 0.549 | 46.41 | 20.0% | 95.7% | 0.157 |  |
| **Post-PCI PLR** | Recorded any adverse event | 229 | 8 | 221 | 0.669 | 13.47 | 75.0% | 61.5% | 0.365 |  |
| **Post-PCI PLR** | Derived composite in-hospital clinical event | 249 | 67 | 182 | 0.636 | 14.43 | 56.7% | 69.8% | 0.265 |  |
| **Post-PCI PLR** | Heart failure | 248 | 18 | 230 | 0.661 | 14.85 | 66.7% | 67.4% | 0.341 | Main post-PCI cutoff used in tables |
| **Post-PCI PLR** | 90-day mortality | 245 | 7 | 238 | 0.800 | 17.7 | 85.7% | 77.3% | 0.630 |  |

## Supplementary Table S3. Significant independent associations from multivariable logistic regression analysis of preprocedural and postprocedural Platelet-to-Lymphocyte Ratio (PLR) with in-hospital clinical outcomes and 90-day mortality

| **Exposure** | **Predictor format** | **Outcome** | **N** | **Events** | **Non-events** | **Adjusted OR (95% CI)** | **p-value** | **Significant** |
| --- | --- | --- | --- | --- | --- | --- | --- | --- |
| **Pre-PCI PLR** | Continuous per 10-unit increase | Derived composite in-hospital clinical event | 260 | 70 | 190 | 1.41 (1.13–1.74) | 0.002* | Yes |
| **Pre-PCI PLR** | ROC cutoff ≥21.68 vs <21.68 | Derived composite in-hospital clinical event | 260 | 70 | 190 | 2.90 (1.55–5.44) | <0.001* | Yes |
| **Post-PCI PLR** | Continuous per 10-unit increase | Derived composite in-hospital clinical event | 238 | 65 | 173 | 1.65 (1.22–2.24) | 0.001* | Yes |
| **Post-PCI PLR** | Continuous per 10-unit increase | Heart failure | 237 | 18 | 219 | 1.58 (1.07–2.31) | 0.02* | Yes |
| **Post-PCI PLR** | Continuous per 10-unit increase | 90-day mortality | 235 | 7 | 228 | 2.46 (1.33–4.54) | 0.004* | Yes |
| **Post-PCI PLR** | ROC cutoff ≥14.85 vs <14.85 | Derived composite in-hospital clinical event | 238 | 65 | 173 | 2.26 (1.21–4.20) | 0.01* | Yes |
| **Post-PCI PLR** | ROC cutoff ≥14.85 vs <14.85 | Heart failure | 237 | 18 | 219 | 3.68 (1.32–10.25) | 0.013* | Yes |
| **Post-PCI PLR** | ROC cutoff ≥14.85 vs <14.85 | 90-day mortality | 235 | 7 | 228 | 10.55 (1.14–98.15) | 0.038* | Yes |

Note: Multivariable logistic regression models were adjusted for age, sex, smoking status, hypertension, diabetes mellitus, dialysis-dependent renal failure, and preprocedural shock. Continuous PLR estimates are reported per 10-unit increase.

## Supplementary Table S4. Statistically significant unadjusted comparisons of clinical and procedural variables stratified by preprocedural Platelet-to-Lymphocyte Ratio (PLR) (< 21.68 vs ≥ 21.68) and postprocedural Platelet to Lymphocyte Ratio (PLR) (< 14.85 vs ≥ 14.85)

| **Source table** | **Comparison** | **Variable** | **Group 1** | **Group 1 value** | **Group 2** | **Group 2 value** | **p-value** |
| --- | --- | --- | --- | --- | --- | --- | --- |
| **Table 1 Pre Baseline** | Baseline characteristics by pre-PCI PLR (<21.68 vs ≥21.68) | Preprocedural troponin, median (IQR) | < 21.68 | 34.1 (9.69, 154.8) | ≥ 21.68 | 99 (17, 1449) | 0.003* |
| **Table 2 Pre Procedural** | Procedural/angiographic characteristics by pre-PCI PLR (<21.68 vs ≥21.68) | First ECG-to-transfer time, min, median (IQR) | < 21.68 | 10 (10, 15) | ≥ 21.68 | 10 (5, 10) | 0.046* |
| **Table 2 Pre Procedural** | Procedural/angiographic characteristics by pre-PCI PLR (<21.68 vs ≥21.68) | Intra-aortic balloon pump use | < 21.68 | 85 (42.3%) | ≥ 21.68 | 42 (60.0%) | 0.011* |
| **Table 3 Pre Outcomes** | Postprocedural labs/outcomes by pre-PCI PLR (<21.68 vs ≥21.68) | Postprocedural creatinine, median (IQR) | < 21.68 | 0.9 (0.73, 1.1) | ≥ 21.68 | 0.91 (0.8, 1.4) | 0.043* |
| **Table 3 Pre Outcomes** | Postprocedural labs/outcomes by pre-PCI PLR (<21.68 vs ≥21.68) | Postprocedural troponin, median (IQR) | < 21.68 | 735 (40.4, 2355.5) | ≥ 21.68 | 2252 (111, 5887) | 0.013* |
| **Table 3 Pre Outcomes** | Postprocedural labs/outcomes by pre-PCI PLR (<21.68 vs ≥21.68) | Life-threatening arrhythmia | < 21.68 | 2 (1.0%) | ≥ 21.68 | 4 (5.9%) | 0.039* |
| **Table 3 Pre Outcomes** | Postprocedural labs/outcomes by pre-PCI PLR (<21.68 vs ≥21.68) | Circulatory support | < 21.68 | 19 (9.5%) | ≥ 21.68 | 13 (18.8%) | 0.040* |
| **Table 3 Pre Outcomes** | Postprocedural labs/outcomes by pre-PCI PLR (<21.68 vs ≥21.68) | Derived composite in-hospital clinical event | < 21.68 | 42 (20.8%) | ≥ 21.68 | 30 (42.9%) | <0.001* |
| **Table 3 Pre Outcomes** | Postprocedural labs/outcomes by pre-PCI PLR (<21.68 vs ≥21.68) | Hospital stay, days, median (IQR) | < 21.68 | 2 (1, 3) | ≥ 21.68 | 3 (2, 4) | <0.001* |
| **Table 3 Pre Outcomes** | Postprocedural labs/outcomes by pre-PCI PLR (<21.68 vs ≥21.68) | Postprocedural LVEF, median (IQR) | < 21.68 | 50 (40, 55) | ≥ 21.68 | 45 (31.2, 55) | 0.037* |
| **Table 4 Post Baseline** | Baseline characteristics by post-PCI PLR (<14.85 vs ≥14.85) | Age, median (IQR) | < 14.85 | 58 (51, 64) | ≥ 14.85 | 60 (54.2, 70) | 0.026* |
| **Table 4 Post Baseline** | Baseline characteristics by post-PCI PLR (<14.85 vs ≥14.85) | Smoking | < 14.85 | 106 (65.0%) | ≥ 14.85 | 40 (46.5%) | 0.005* |
| **Table 4 Post Baseline** | Baseline characteristics by post-PCI PLR (<14.85 vs ≥14.85) | Previous diagnosed arrhythmia | < 14.85 | 0 (0.0%) | ≥ 14.85 | 4 (4.7%) | 0.014* |
| **Table 4 Post Baseline** | Baseline characteristics by post-PCI PLR (<14.85 vs ≥14.85) | Preprocedural LVEF, median (IQR) | < 14.85 | 50 (40, 60) | ≥ 14.85 | 43.5 (33.5, 50) | <0.001* |
| **Table 4 Post Baseline** | Baseline characteristics by post-PCI PLR (<14.85 vs ≥14.85) | Preprocedural troponin, median (IQR) | < 14.85 | 31.2 (6.57, 153.7) | ≥ 14.85 | 75 (15, 1095) | 0.004* |
| **Table 5 Post Procedural** | Procedural/angiographic characteristics by post-PCI PLR (<14.85 vs ≥14.85) | Residual distal stenosis >50% | < 14.85 | 49 (30.1%) | ≥ 14.85 | 37 (43.0%) | 0.041* |
| **Table 6 Post Outcomes** | Postprocedural labs/outcomes by post-PCI PLR (<14.85 vs ≥14.85) | Postprocedural creatinine, median (IQR) | < 14.85 | 0.88 (0.74, 1.04) | ≥ 14.85 | 0.95 (0.78, 1.36) | 0.041* |
| **Table 6 Post Outcomes** | Postprocedural labs/outcomes by post-PCI PLR (<14.85 vs ≥14.85) | Postprocedural troponin, median (IQR) | < 14.85 | 447.5 (35.8, 1902.5) | ≥ 14.85 | 1927 (72.5, 5308.8) | 0.003* |
| **Table 6 Post Outcomes** | Postprocedural labs/outcomes by post-PCI PLR (<14.85 vs ≥14.85) | Mechanical ventilation | < 14.85 | 4 (2.5%) | ≥ 14.85 | 10 (11.8%) | 0.007* |
| **Table 6 Post Outcomes** | Postprocedural labs/outcomes by post-PCI PLR (<14.85 vs ≥14.85) | Circulatory support | < 14.85 | 14 (8.8%) | ≥ 14.85 | 18 (21.2%) | 0.006* |
| **Table 6 Post Outcomes** | Postprocedural labs/outcomes by post-PCI PLR (<14.85 vs ≥14.85) | Derived composite in-hospital clinical event | < 14.85 | 34 (20.9%) | ≥ 14.85 | 33 (38.4%) | 0.003* |
| **Table 6 Post Outcomes** | Postprocedural labs/outcomes by post-PCI PLR (<14.85 vs ≥14.85) | Cardiogenic shock | < 14.85 | 3 (1.9%) | ≥ 14.85 | 11 (12.8%) | <0.001* |
| **Table 6 Post Outcomes** | Postprocedural labs/outcomes by post-PCI PLR (<14.85 vs ≥14.85) | Heart failure | < 14.85 | 7 (4.3%) | ≥ 14.85 | 11 (12.8%) | 0.014* |
| **Table 6 Post Outcomes** | Postprocedural labs/outcomes by post-PCI PLR (<14.85 vs ≥14.85) | Hospital stay, days, median (IQR) | < 14.85 | 2 (1, 3) | ≥ 14.85 | 2 (2, 4) | 0.001* |
| **Table 6 Post Outcomes** | Postprocedural labs/outcomes by post-PCI PLR (<14.85 vs ≥14.85) | Postprocedural LVEF, median (IQR) | < 14.85 | 50 (43, 60) | ≥ 14.85 | 40 (30, 55) | <0.001* |
| **Table 6 Post Outcomes** | Postprocedural labs/outcomes by post-PCI PLR (<14.85 vs ≥14.85) | Death within 90 days | < 14.85 | 1 (0.6%) | ≥ 14.85 | 6 (7.1%) | 0.007* |
